# KDELC2 Upregulates Glioblastoma Angiogenesis via Reactive Oxygen Species Activation and Tumor-Associated Macrophage Proliferation

**DOI:** 10.3390/antiox12040923

**Published:** 2023-04-13

**Authors:** Yu-Ling Tsai, Ying Chen, Ying-Chuan Chen, Wen-Chiuan Tsai

**Affiliations:** 1 Department of Pathology, Tri-Service General Hospital, National Defense Medical Center, Taipei 114, Taiwan; 2 Department of Biology and Anatomy, National Defense Medical Center, Taipei 114, Taiwan; 3 Department of Physiology and Biophysics, National Defense Medical Center, Taipei 114, Taiwan

**Keywords:** KDELC2, glioblastoma, angiogenesis, ROS, TAM, ER stress, THP-1, macrophage differentiation, HUVEC, tube formation

## Abstract

Glioblastoma is notorious for its rapid progression and neovascularization. In this study, it was found that KDEL (Lys-Asp-Glu-Leu) containing 2 (KDELC2) stimulated vasculogenic factor expression and induced human umbilical vein endothelial cell (HUVEC) proliferation. The NLRP3 inflammasome and autophagy activation via hypoxic inducible factor 1 alpha (HIF-1α) and mitochondrial reactive oxygen species (ROS) production was also confirmed. The application of the NLRP3 inflammasome inhibitor MCC950 and autophagy inhibitor 3-methyladenine (3-MA) indicated that the above phenomenon activation correlated with an endothelial overgrowth. Furthermore, KDELC2 suppression decreased the endoplasmic reticulum (ER) stress factors’ expression. The ER stress inhibitors, such as salubrinal and GSK2606414, significantly suppressed HUVEC proliferation, indicating that ER stress promotes glioblastoma vascularization. Finally, shKDELC2 glioblastoma-conditioned medium (CM) stimulated TAM polarization and induced THP-1 cells to transform into M1 macrophages. In contrast, THP-1 cells co-cultured with compensatory overexpressed (OE)-KDELC2 glioblastoma cells increased IL-10 secretion, a biomarker of M2 macrophages. HUVECs co-cultured with shKDELC2 glioblastoma-polarized THP-1 cells were less proliferative, demonstrating that KDELC2 promotes angiogenesis. Mito-TEMPO and MCC950 increased caspase-1p20 and IL-1β expression in THP-1 macrophages, indicating that mitochondrial ROS and autophagy could also interrupt THP-1-M1 macrophage polarization. In conclusion, mitochondrial ROS, ER stress, and the TAMs resulting from OE-KDELC2 glioblastoma cells play important roles in upregulating glioblastoma angiogenesis.

## 1. Introduction

Glioblastoma, the highest World Health Organization (WHO) grade of gliomas, is notoriously aggressive and drug-resistant [1]. Histologically, the most striking difference between high- and low-grade gliomas is the density of tumor blood vessels [1]. Angiogenesis is the growth of new capillaries from preexisting vascular endothelial cells [2], and it maintains glioma stem cells and promotes tumor progression [3]. Several transcription factors, including vascular endothelial growth factor (VEGF), platelet-derived growth factor (PDGF), transforming growth factor-β (TGF-β), and fibroblast growth factors (FGFs), are activated in tumor angiogenesis [4,5,6]. Bevacizumab, an anti-VEGF monoclonal antibody, was considered a potential inhibitor of glioblastoma progression, but it failed to improve overall survival in phase III trials [7]. Previous studies provided evidence of “vasculogenic mimicry,” in which some tumor endothelial cells are derived from glioblastoma or cancer stem cells in an oxygen-deficient microenvironment [8,9,10]. Compared to low-grade gliomas, these tumor-derived endothelial cells have different angiogenic mediators, such as hypoxic inducible factor 1 alpha (HIF-1α), neural cell adhesion molecule, FGF, and angiopoietin receptor Tie-2 [11]. The stabilization of HIF-1α in a hypoxic cellular condition depends on the generation of glioblastoma mitochondrial reactive oxygen species (ROS) [12]. The mitochondrial ROS cascade also regulates endothelial metabolism [13].

Of all the Notch signaling-related factors, KDELC2 and KDELC1 are responsible for modifying the *O*-linked glycans on Notch 1 epidermal growth factor-like repeat 11 (EGF11) and Notch 3 EGF10 [14]. KDELC2 is essential for Notch 1–4 receptor development and Notch cascade activation [15]. Kofier et al. [16] showed that Notch signaling can induce tumor angiogenesis via tumor-associated macrophage (TAM) recruitment, inflammatory cytokine activation, and the connection between Notch receptors and delta-like canonical Notch Ligand 4 (DLL4) ligands. In the previous study, KDELC2 induced glioblastoma aggressive behaviors, including tumor proliferation, migration, invasion, stemness, and angiogenesis [15]. Additionally, the decrease in glioblastoma angiogenesis after KDELC2 knockdown depended on the low expression of VEGFA, VEGFR1, and CD31 [15]. Recent evidence indicated the anti-angiogenic therapeutic regimen targeting angiogenic factors failed to show unsatisfactory survival benefits [17]. The stimulation of other compensatory signaling pathways of neovasculogenesis might be a possible explanation. Unfortunately, the detailed mechanism involving the above angiogenic factors and KDELC2 expression remains undetermined.

This study demonstrated that KDELC2 could stimulate glioblastoma angiogenesis by activating ROS, endoplasmic reticulum (ER) stress, and TAMs. In addition, the nucleotide-binding domain leucine-rich family pyrin-containing 3 (NLRP3) inflammasome and autophagy were important in promoting glioblastoma vascularization induced by mitochondrial ROS. Furthermore, our results showed the interaction of KDELC2-induced TAMs with ROS and the NLRP3 inflammasome. To our knowledge, this is the first study to confirm the mechanism by which KDELC2 promotes the neovascularization of glioblastoma.

## 2. Materials and Methods

### 2.1. In Silico Study

The data for KDELC2 mRNA expression microarrays and attendant WHO tumor grades for samples were obtained from the Chinese Glioma Genome Atlas (CGGA) database (http://www.cgga.org.cn/ (accessed on 10 January 2023)).

### 2.2. Cell Cultures

The human glioma cell lines GBM8401, U87MG, and T98G were obtained from the American Type Culture Collection (Rockville, MD, USA). GBM8401 cells expressed mutant TP53 and MGMT methylation, U87MG expressed wild-type TP53 with MGMT methylation, while T98G cells expressed mutant TP53 with MGMT overexpression. GBM8401 (8 × 10^5^/10 cm tissue culture dish) and U87MG (1.2 × 10^6^/10 cm tissue culture dish) cells were maintained in Dulbecco’s modified eagle’s medium (DMEM, Gibco, Grand Island, NY, USA) supplemented with 10% heat-inactivated fetal bovine serum (FBS; Gibco) and 1% Penicillin-Streptomycin (Gibco). T98G cells (1 × 10^6^/10 cm tissue culture dish) were maintained in a Minimal Essential Medium (MEM, Gibco) with 10% heat-inactivated FBS, 1% Penicillin-Streptomycin, and 2 mM L-glutamine. Human umbilical vein endothelial cells (HUVECs) (3 × 10^5^/10 cm tissue culture dish) were maintained in an endothelial cell medium (ScienCell, San Diego, CA, USA). Human THP-1 monocytes (4 × 10^6^/10 cm tissue culture dish) were obtained from the American Type Culture Collection. To induce monocyte-to-macrophage differentiation, THP-1 cells (2 × 10^6^/6 cm tissue culture dish) were cultured for 24 h in RPMI-1640 medium supplemented with 100 nM phorbol 12-myristate 13-acetate (Sigma-Aldrich, St. Louis, MO, USA) [18]. All cells were maintained in the incubator (Thermo Fisher Scientific, Waltham, MA, USA) with 5% CO2 and at 37 °C.

### 2.3. Stable Expression Clones of shRNAs

The knockdown lentiviral vectors of KDELC2 (#180: GCCAAGTTGATGGGTTTCTTT; #220: CCGGAGATCTTTAGGGAAATA) and luciferase-specific shRNA (shLuc: GCGGTTGCCAAGAGGTTCCAT) as the control were purchased from the National RNAi Core Facility (Academia Sinica, Taipei, Taiwan). GBM8401 or U87MG cells were infected with lentivirus-bearing KDELC2-specific shRNA or luciferase-specific shRNA, respectively, and incubated with puromycin (Invitrogen, Carlsbad, CA, USA) to select stably infected cells. The knockdown efficiency was evaluated using real-time quantitative reverse transcription-polymerase chain reaction (qRT-PCR) and Western blotting.

### 2.4. Overexpression of KDELC2

The overexpression plasmids were synthesized by Sino Biological (Houston, TX, USA). GBM8401 or T98G cells were transfected with plasmids using the jetPRIME^®^ reagent (Polyplus, New York, NY, USA) in an antibiotic-free Opti-MEM medium (Gibco) according to the manufacturer’s protocol.

### 2.5. RNA Isolation and qRT-PCR

The total RNA was extracted using TriZOL reagent (Invitrogen), and cDNA was prepared according to the manufacturer’s protocol (Bioline, Meridian Bioscience, Cincinnati, OH, USA). The qRT-PCR assay was performed following the previous protocol [15]. All specific primer pairs are listed in Table S1.

### 2.6. ROS Detection

The production of intracellular ROS in U87MG, GBM8401, and HUVECs was determined by measuring the fluorescence intensity of 2′,7′-dichlorofluorescein and the oxidation product of 2′,7′-dichlorofluorescein diacetate (Invitrogen). The cells (1 × 10^5^/mL in a 96-well plate) were incubated with 2′,7′-dichlorofluorescein diacetate for 30 min. The fluorescence intensity of 2′,7′-dichlorofluorescein was detected at an excitation wavelength of 485 nm and an emission wavelength of 530 nm using a microplate absorbance reader. The cells were stained with MitoSOX (Invitrogen) to evaluate mitochondrial ROS generation. The fluorescence intensity of MitoSOX was detected at an excitation wavelength of 510 nm and an emission wavelength of 580 nm using a microplate absorbance reader. The methods of evaluating intracellular and mitochondrial ROS were similar.

### 2.7. Western Blot Analysis

The cells seeded in a 10 cm tissue culture dish (1 × 10^6^) were incubated for 24 h, after which salubrinal (20 μM), GSK2606414 (1 μM), N-acetyl cysteine (NAC, 10 mM), MCC950 (1 μM), or Mito-TEMPO (500 μM) was added for 24 h. The culture medium was removed, and the cells were washed with ice-cold PBS and then extracted using a RIPA buffer (Phosphatase Inhibitor Cocktail, Protease Inhibitor Cocktail, 1 mM Na 3VO4, 1 μg/mL of leupeptin, and 1 mM PMSF). The lysate was centrifuged at 12,000× *g* at 4 °C for 10 min. The supernatant and quantified protein concentration was collected by using a protein assay kit (Bio-rad, Hercules, CA, USA). The sample buffer (50% glycerol, 10% sodium dodecyl sulfate, 0.05% β-mercaptoethanol, 0.5% Bromophenol blue, 250 mM Tris-HCL, and pH 6.8) and lysis buffer were added in different amounts based on different protein concentrations. The protein lysates (100 μg) were run on 10% or 15% SDS-PAGE gels. After being stacked at 70 V, separated at 120 V, and electrotransferred to a PVDF membrane at 300 mA for 1 h, the membrane was blocked by incubation for 30 min at room temperature in a blocking buffer (5% BSA in PBS) with primary antibodies at 4 °C overnight. After six washes in PBST for 10 min, the membrane was incubated for 1 h at room temperature with an appropriate HRP-conjugated secondary antibody diluted in a blocking buffer. The membrane-bound antibody detected was incubated with Enhanced Reagent Plus (PerkinElmer, Waltham, MA, USA), and the PVDF membranes were scanned with a UVP BioSpectrum Imaging System (Financial HealthCare). All above-mentioned antibodies are listed in Table S2.

### 2.8. Enzyme-Linked Immunosorbent Assay (ELISA)

The principle of sandwich ELISA was performed to detect the level of VEGFA (DY293B), IL-1β (DY201), FGF (DY233), MCP-1 (DY279), IL-10 (DY217B), TNF-α(DY210), CD38 (DY2404-05), IL12 (DY1270), and IL6 (DY206) expression. Initially, the supernatants of the cultured cells were measured using ELISA kits (R&D Systems). In brief, 100 μL of capture antibody solution was added to each well of a 96-well microplate (Corning, Corning, NY, USA) and incubated overnight at room temperature. After repeatedly washing the plate with a washing buffer (0.05% Tween 20 in PBS) and adding a reagent diluent (1% BSA in PBS) with further appropriate incubation, 100 μL of the detection antibody and streptavidin-HRP were added as the secondary antibody and enzyme-specific substrate, respectively. After adding 50 μL stop solution to each well, the absorbance at 450 nm of each well was measured using a microplate reader.

### 2.9. Tube Formation

The tube formation (the ability of cellular angiogenesis) was measured on an extracellular matrigel in a 96-well plate. Briefly, the matrigel was thawed on ice overnight. A total of 50 μL of matrigel (#356231, Corning) was added per well (96-well plate) and allowed to polymerize for 30 min at 37 °C. The HUVECs were trypsinized and counted and they were seeded (1 × 10^4^) onto the matrigel in 100 μL of endothelial cell medium (EBM, Sciencell, CA, USA) with 1% FCS and treated with 50 μL of supernatants from the cultured GBM8401, U87MG, T98G, and THP-1. After 16 h, tubular structures were randomly imaged using a phase-contrast microscope at 100× magnification. The tube formation was analyzed by vessel morphometric parameters, and the vessel’s length was measured using AngioTool software. The experiments were reproduced 3 times independently.

### 2.10. Orthotopic Xenograft Animal Model

Female BALB/c AnN.Cg-Foxnlnu/CrlNarl mice (8 weeks old) were purchased from the National Laboratory Animal Center, Taipei, Taiwan. One week later, the mice were anesthetized and placed in a stereotactic frame with GBM8401-Luc cells (1 × 10^5^) in 4 μL 50% matrigel (PBS diluent) using a 26-gauge needle at the right cerebral hemisphere and 3 mm below the dura. The animals were randomly assigned to two groups: one received shLuc, and the other received shKDELC2. After 9 days, the mice were euthanized with mixed tiletamine and zolazepam (1:1) and xylazine, and their brains were fixed in 10% formalin, embedded in paraffin, and cut into serial sections. The brain tissues were sliced into 3-μm-thick sections. A histological evaluation was performed by hematoxylin and eosin staining. An immunohistochemical stain was performed by a Ventana BenchMark ULTRA system (Roche, Basel, Switzerland). The primary antibodies were diluted in Primary Antibody Dilute (ScyTek, Logan, UT, USA). The expression of the aforementioned proteins was examined, and the images were captured in each group by light microscopy.

### 2.11. Statistical Analysis

The data are representative of at least three independent experiments, and the results are expressed as the means ± SEM for the total number of experiments. GraphPad Prism 5.0 software was used to analyze the data. The Kruskal–Wallis test was used to analyze the differences. The Mann–Whitney U test was performed for a post hoc analysis.

## 3. Results

### 3.1. KDELC2 Expression Correlates with Glioma Tumor Grade and Glioblastoma Neovascularization

**Figure 1:**
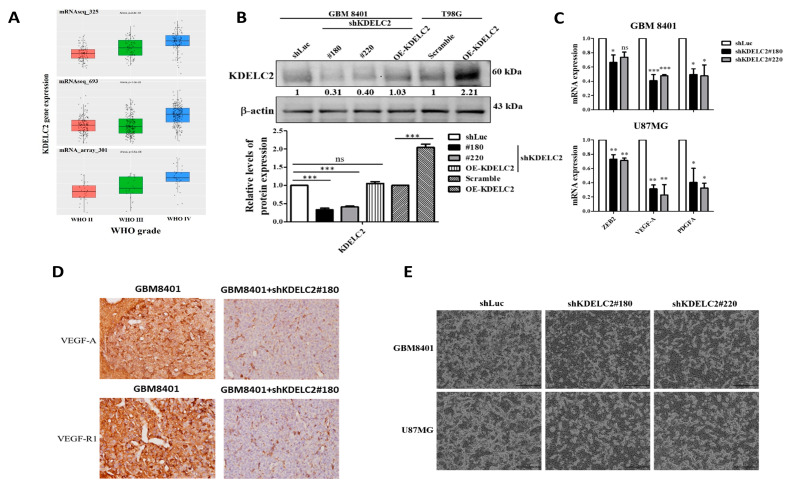
Expression analysis of KDELC2 in glioblastomas. (**A**) High KDELC2 mRNA expression correlated with advanced gliomas in the CGGA database. (**B**) The shKDELC2-transfected GBM8401 suppressed KDELC2 protein expression, but the compensated OE-KDELC2 transfection reversed low KDELC2 expression. OE-KDELC2 T98G had the highest expression. (**C**) Low ZEB2, VEGF-A, and PDGFA mRNA expression in GBM8401 and U87MG glioblastoma cells with shKDELC2 transfection. (**D**) The transfection of shKDELC2 of GBM8401 decreased VEGF-A and VEGF-R1 expression in the orthotopic xenograft animal model. (**E**) HUVECs with supernatants from cultured GBM8401 and U87MG with shKDELC2 transfection suppressed tube lengths and nodes. Bars, means ± SEM. *, *p* < 0.05; **, *p* < 0.01; ***, *p* < 0.001; ns—non-significant.

The CGGA data showed that a higher KDELC2 mRNA expression positively correlated with more advanced glioma tumor grades (Figure 1A). In addition, we observed that glioblastoma cells significantly increased the KDELC2 expression compared to low-grade glioma cells. There was a decrease in the KDELC2 mRNA expression after shKDELC2#180 and #220 transfection, confirming a KDELC2 knockdown (Figure 1B). Furthermore, zinc finger E-Box binding homeobox 2 (ZEB2), VEGF-A, and PDGFA mRNA expression was significantly lower after KDELC2 knockdown in both GBM8401 and U87MG cells (Figure 1C). The differences in angiogenic factor expressions after KDELC2 knockdown were detected in orthotropic human glioblastoma xenograft mouse models (Figure 1D). In the HUVEC tube formation study, less branching and node growth were observed in shKDELC2-transfected glioblastoma cells (Figure 1E). Our results indicated that KDELC2 suppression in glioblastoma cells significantly inhibited tumor angiogenesis. Therefore, we concluded that KDELC2 promotes glioblastoma angiogenesis by upregulating ZEB2, VEGF-A, VEGF-R1, and PDGFA expression.

### 3.2. KDELC2 Induces Angiogenesis by Elevating Mitochondrial ROS in Glioblastoma Cells and HUVECs

**Figure 2:**
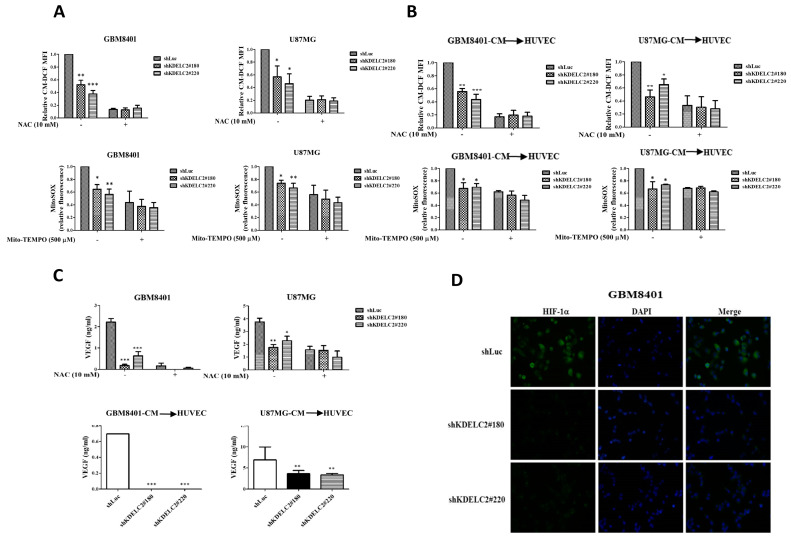
KDELC2 knockdown inhibited angiogenesis in glioblastoma. (**A**) Low levels of cellular and mitochondrial ROS were observed in shKDELC2-transfected GBM8401 and U87MG cells, but no difference in ROS levels was observed after applying ROS inhibitors. (**B**) Low levels of ROS were identified in HUVEC co-cultured with shKDELC2-transfected GBM8401 and U87MG-CM. (**C**) GBM8401 and U87MG with transfected shKDELC2 presented low VEGF-A and VEGFR1 expression, but the application of NAC revealed no difference in the above pro-angiogenic factors. The HUVEC co-cultured with shKDELC2 glioblastoma cells had similar results. (**D**) The shKDELC2-transfected glioblastoma cells had a low expression of HIF-1α. Bars, means ± SEM. *, *p* < 0.05; **, *p* < 0.01; ***, *p* < 0.001.

Both cellular and mitochondrial ROS play important roles in cancer development and tumor microvascular proliferation. Cellular and mitochondrial ROS detection assays were performed to determine whether KDELC2 expression activated ROS in glioblastoma cells. This study showed that shKDELC2-transfected GBM8401 and U87MG cells had lower intracellular ROS levels than the shLuc-transfected glioblastoma cells (Figure 2A). As N-acetylcysteine (NAC) is a cellular ROS scavenger, no significant difference in cellular ROS levels was detected between the KDELC2 knockdown groups of the above glioblastoma cells and other cells after NAC application (Figure 2A). Similarly, compared to the shLuc group of tumors, mitochondrial ROS levels significantly decreased in shKDELC2-transfected GBM8401 and U87 glioblastoma cells, but there was no statistical difference after mitochondria-targeted antioxidant (Mito-TEMPO) administration (Figure 2A).

When exploring the impact of ROS activation on HUVECs after adding shKDELC2 glioblastoma-CM, we found a significant decrease in cellular and mitochondrial ROS levels in the HUVECs with shKDELC2 glioblastoma-CM (Figure 2B). To determine the effect of KDELC2-induced ROS on tumor angiogenesis, we evaluated the VEGF expression in glioblastoma cells with or without the addition of NAC. Our results revealed no significant change in VEGF expression between the shLuc- and shKDELC2-transfected glioblastoma cells after NAC administration (Figure 2C), implying that KDELC2 inhibition could downregulate the angiogenic factor expression. Furthermore, the immunofluorescence results revealed that the shKDELC2-transfected GBM8401 cells had a lower HIF-1α expression than the shLuc-transfected glioblastoma cells (Figure 2D). In conclusion, we discovered the role of KDELC2 in inducing glioblastoma angiogenesis via the activation of ROS and HIF-1α. Additionally, a decreased HIF-1α expression after KDELC2 suppression implied that KDELC2 expression induces glioblastoma tumorigenesis under nutrient-deprived conditions.

### 3.3. Upregulation of ER Stress and HIF-1α Expression by KDELC2 Overexpression in Glioblastoma Cells

**Figure 3:**
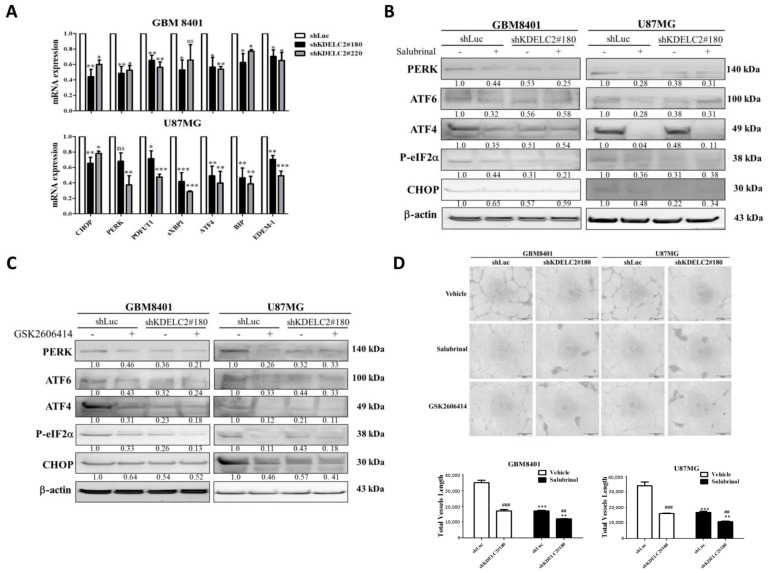
KDELC2 suppression decreased the level of ER stress in glioblastoma. (**A**) The shKDELC2-transfected GBM8401 and U87MG inhibited ER stress-related factor expression. (**B**) The application of salubrinal resulted in no difference in the ER stress in shKDELC2-transfected glioblastoma cells. (**C**) The administration of GSK2606414 in shKDELC2-transfected glioblastoma cells revealed no difference in ER stress-related factors expression. (**D**) The addition of salubrinal and GSK2606414 in shLuc and shKDELC2 both decreased HUVEC proliferation. Bars, means ± SEM. *, *p* < 0.05; **, *p* < 0.01; ***, *p* < 0.001; ^##^, *p* < 0.01; ^###^, *p* < 0.001; ns—non-significant.

The ER stress is induced by the accumulation of misfolded proteins causing an unfolded protein response [19]. In this study, we observed that KDELC2 knockdown inhibited C/EBP homologous protein (CHOP) and protein kinase RNA-like endoplasmic reticulum kinase (PERK), spliced X-box-binding protein 1 (sXBP1), and activated transcription factor 4 (ATF4), binding immunoglobulin protein (BIP) and ER-degradation-enhancing alpha-mannosidase-like protein 1 (EDEM1) mRNA expression (Figure 3A). We then used salubrinal and GSK2606414 as ER stress inhibitors and suppressed PERK, ATF4, ATF6, and phosphorylated eukaryotic initiation factor-2α (p-eIF-2α) expression (Figure 3B,C). Compared to the glioblastoma group with the ER stress activated by KDELC2, no difference in HUVEC proliferation was found after applying salubrinal and GSK2606414 (Figure 3D). Therefore, we confirmed KDELC2 resulted in the activation of an ER stress that stimulated glioblastoma angiogenesis. Since overexpressed ER stress-related factors, including PERK, ATF4, and CHOP, upregulate cytosolic and mitochondrial ROS [20], the activation of mitochondrial ROS by KDELC2-enhanced ER stress might be an explanation, but more data are needed to confirm this.

### 3.4. KDELC2 Overexpression Activates NLRP3 Inflammasome and Upregulates Glioblastoma Autophagy via Increased Mitochondrial ROS Production

**Figure 4:**
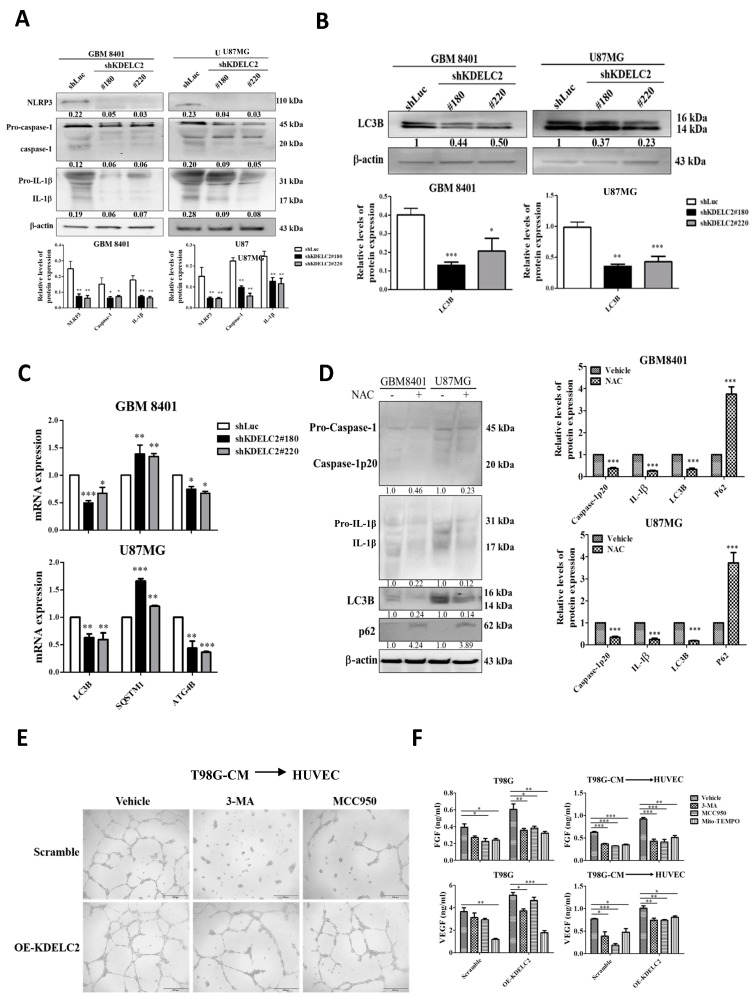
The interaction of KDELC2 with the activation of NLRP3 inflammasome and autophagy in glioblastoma cells. (**A**) The decrease in NLRP3, caspase-1, and IL-1β protein expression after shKDELC2-transfected GBM8401 and U87MG. (**B**) Western blot analysis revealed decreased expression of LC3β in shKDELC2-transfected GBM8401 and U87MG. (**C**) KDELC2 knockdown glioblastoma led to a relatively lower mRNA expression of LC3β, STSQM1 (p62), and ATG4B. (**D**) The administration of NAC in glioblastoma cells inhibited the activated caspase-1, IL-1β, LC3β, and p62 protein expression. (**E**) The suppression of HUVEC proliferation was detected in glioblastoma cells after the use of 3MA and MCC950. (**F**) The application of 3MA, MCC950, and Mito-tempo inhibited FGF and VEGF expression in T98 and THP-1 co-cultured with T98-CM. Bars, means ± SEM. *, *p* < 0.05; **, *p* < 0.01; ***, *p* < 0.001.

The activation of the NLRP3 inflammasome is associated with glioma cell proliferation, migration, and metastasis [21]. The activated NLRP3 inflammasome includes the nucleotide-binding and oligomerization domain (NOD)-like receptor, adapter apoptosis-associated speck-like protein, and caspase 1, and it is regulated by interleukin-1β (IL-1β) [22]. After the shKDELC2 transfection of the glioblastoma cells, there was a significant decrease in NLRP3, caspase-1, and IL-1β expression compared to the shLuc-transfected GBM8401 and U87 cells (Figure 4A). Therefore, our data confirmed KDELC2 could induce an NLRP3 inflammasome in glioblastoma cells.

Hypoxia-mediated autophagy plays a role in chemo-resistance to anti-angiogenic pharmacologic treatment [23]. Furthermore, mitochondrial ROS-induced autophagy has dual roles in cancers via different signaling pathways, including cytoprotective or programmed cell death pathways [24]. This study evaluated the relationship between KDELC2 suppression and autophagy-related factor expression in glioblastoma. According to the Western blot results and qRT-PCR assays, the shKDELC2-transfected GBM8401 and U87 cell lines had a lower microtubule-associated protein 1A/1B light chain 3B (LC3B) mRNA and protein expression than the shLuc-transfected cell lines (Figure 4B). Furthermore, compared to the shLuc-transfected glioblastoma cells, lower ATG4B and higher p62 mRNA expressions were noted in the shKDELC2-transfected GBM8401 and U87 glioblastoma cells (Figure 4C). The above evidence confirmed that KDELC2 played an important role in the stimulation of autophagy.

We evaluated the inflammasome-related factor expression in the KDELC2 control and overexpressed groups of glioblastoma cells after applying NAC to explore the possible role of mitochondrial ROS in activating the inflammasome. Our results revealed that mitochondrial ROS are critical in inflammasome activation (Figure 4D). Hasan et al. [24] showed that cancer stromal cells can induce autophagy by increasing ROS production in a hypoxic tumor microenvironment (TME). We evaluated the autophagy-related factor expression after NAC administration to investigate mitochondrial ROS-induced glioblastoma autophagy. We found no significant differences in LC3B, p62, and ATG4B expression between the shLuc and OE-KDELC2 glioblastoma groups (Figure 4D). Our results implied that the stimulation of glioblastoma autophagy depended on KDELC2-induced ROS activation.

As the inflammasome and autophagy can induce endothelial progenitor cell hyperplasia, we surveyed the association of the KDELC2-related inflammasome and autophagy with glioblastoma microvascular proliferation after applying the NLRP3 inflammasome inhibitor MCC950 and autophagy inhibitor 3-methyladenine (3-MA). The HUVECs showed a significant decrease in endothelial proliferation after MCC950 and 3-MA application (Figure 4E). Similarly, no significant difference in VEGF and FGF mRNA expression was found in T98G cells after the addition of MCC950 and 3-MA between the control and OE-KDELC2 groups of glioblastoma (Figure 4F). We concluded that KDELC2 might stimulate glioblastoma angiogenesis by activating the NLRP3 inflammasome and autophagy via mitochondrial ROS generation.

### 3.5. KDELC2 Stimulates the Transformation of M2 Macrophages and Promotes Tumor Angiogenesis

**Figure 5:**
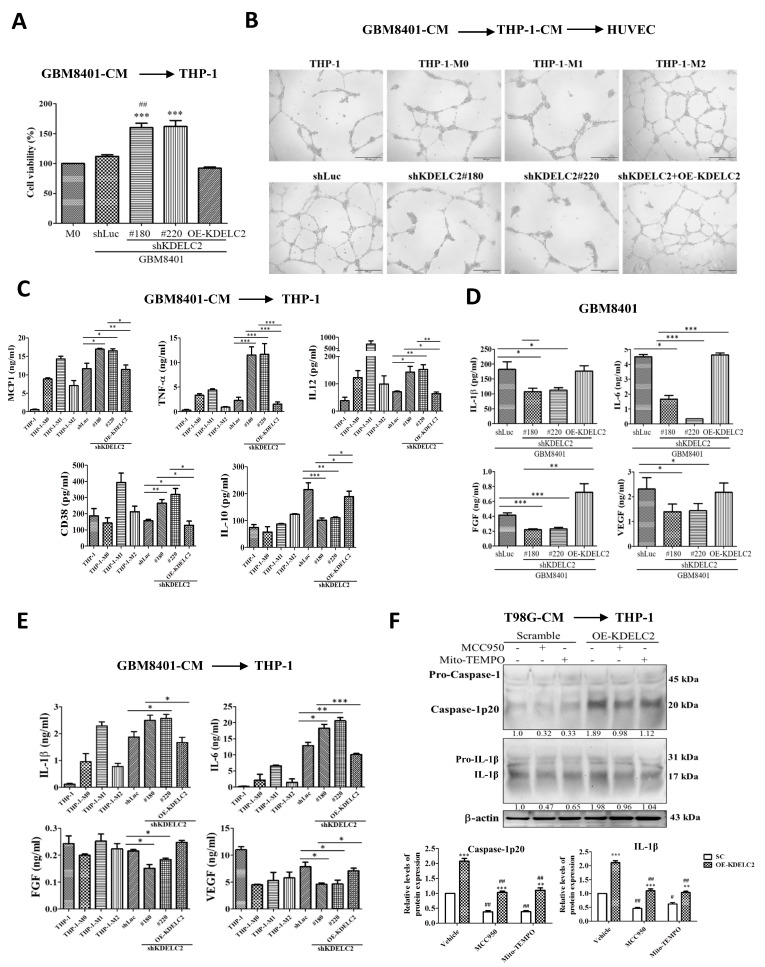
KDELC2 induced the polarization of THP-1. (**A**) Knockdown of KDELC2 of glioblastoma cells had favorable viability of THP-1 macrophages. (**B**) The degree of the HUVEC proliferation of shKDELC2 and compensated OE-KDELC2 glioblastoma cells was similar to THP-1-M1 and THP-1-M2 cells, respectively. (**C**) High expression of MCP-1, TNF-α, IL-12, and CD38 was noted in shKDELC2 glioblastoma cells, but relatively high IL-10 expression was observed in shLuc and compensated OE-KDELC2 glioblastomas. (**D**) Low expression of IL-1β, IL-6, FGF, and VEGF was noted in shKDELC2 transfected glioblastoma cells. (**E**) High IL-1β and IL-6, but low FGF and VEGF expression in THP-1 macrophages co-cultured with shKDELC2 transfected glioblastoma-CM. (**F**) The application of MCC950 and Mito-TEMPO significantly increased caspase-1p20 and IL-1β in THP-1 macrophages with shKDELC2-transfected glioblastoma-CM. Bars, means ± SEM. *, *p* < 0.05; **, *p* < 0.01; ***, *p* < 0.001; ^#^, *p* < 0.05; ^##^, *p* < 0.01; ns—non-significant.

TAMs are a part of the TME and respond to tumor growth and chemo-resistance by dysregulating anti-tumor T cell immunity [25]. M1 macrophages strengthen tumor cytotoxicity by activating nitric oxide synthases (NOS), ROS, and IL12. M2 macrophages promote tumor angiogenesis in hypoxic areas by coordinating IL-1, TNF-α, and VEGF [26]. To evaluate the relationship between KDELC2 and TAMs, we detected THP-1 viability after adding GBM8401-CM with OE-KDELC2 or shKDELC2 transfection. The administration of GBM8401-CM to THP-1 transformed them into THP-1-M0 macrophages. Moreover, shKDELC2 glioblastoma cells had significantly higher viability of THP-1 cells than the shLuc group (Figure 5A). However, compared to shLuc-transfected glioblastoma-CM, no significant change in the viability of THP-1 was identified in groups of initially shKDELC2-transfected glioblastoma cells with the overexpressed KDELC2 (Figure 5A).

We also used GBM8401-CM to stimulate THP-1 cells and then performed a tube formation analysis after HUVECs were co-cultured with THP-1-CM to explore the association of TAM polarization and HUVEC proliferation. HUVECs co-cultured with THP-1 via shKDELC2 glioblastoma-CM stimulation decreased the proliferative ability of the endothelial cells significantly, similarly to the HUVECs co-cultured with THP-1-M1 (Figure 5B). Furthermore, the co-culture with THP-1-M2-CM increased the length and node number of the HUVECs more than THP-1-M1, indicating that the M2 TAMs mainly responded to endothelial cell hyperplasia (Figure 5B). Furthermore, the degree of HUVEC proliferation by co-culturing with shLuc and shKDELC2 with the compensatory OE-KDELC2 groups of GBM8401-CM resembled HUVEC co-cultured with THP-1-M2 macrophages (Figure 5B). Therefore, we inferred that KDELC2 suppression elevates TAM viability but mostly tended to differentiate into M1 macrophages and suppressed glioblastoma vascularization.

We also demonstrated that KDELC2 knockdown induced monocyte chemoattractant protein 1 (MCP-1, current name CCL2), TNF-α, interleukin-12 (IL-12), and CD38 mRNA expression but reduced IL-10 mRNA expression. However, the transfection of compensatory KDELC2 could reverse the above effects, implying that KDELC2 could induce the polarization of TAMs to M2 macrophages (Figure 5C). Our results showed lower IL-1β and IL-6 levels in the shKDELC2-transfected glioblastoma cells compared to the shLuc-transfected glioblastoma cells but demonstrated a marked increase in the above factors after the addition of recombinant OE-KDELC2 (Figure 5D). As IL-1β and IL-6 are associated with glioblastoma angiogenesis, we confirmed that KDELC2 suppression could inhibit the expression of some angiogenic factors, such as FGF and VEGF (Figure 5D). In contrast, adding the KDELC2 knockdown GBM8401-CM co-cultured with THP-1 showed higher IL-1β and IL-6 expression and lower FGF and VEGF expression, similarly to THP-1-M1 expression (Figure 5E). Therefore, we concluded that suppressing KDELC2 downregulates glioblastoma angiogenesis by decreasing the production of IL-1β and IL-6 tumor cells, but the above two interleukins secreted by the THP-1-M1 macrophages could inhibit FGF and EGFR expression.

We evaluated the interaction between the growth of THP-1-M1 with mitochondrial ROS and the NLRP3 inflammasome. Compared to the THP-1 cells, a higher caspase-1p20 and IL-1β expression was identified after the application of either MCC950 or Mito-TEMPO to the THP-1 cells co-cultured with OE-KDELC2 T98G-CM (Figure 5F). In conclusion, our results indicated that the mitochondrial ROS and NLRP3 inflammasome effectively downregulated the number of THP-1-M1 macrophages.

## 4. Discussion

ROS represents one kind of oxidative stress and causes mitochondrial damage and free radical generation via xenobiotic drug metabolism, ionization, and carcinogens [27,28]. The different ROS levels result in different cell fates [29,30]. A low level of ROS promotes cell progression and proliferation, but a persistent high dose of ROS might induce cell apoptosis or survival depending on several biological factors. The reaction of the ROS hydroxyl groups with some chromatin protein purines and pyrimidines results in genomic instability and mutated oncogene overexpression via ROS activation [31]. The increased expression of the mutated *Ras* and *p53* oncogenes commonly involves ROS in high-grade gliomas [32,33]. Glioblastoma neoangiogenesis resulting from changes in EGFR, PDGFR, and VEGFR expression is commonly associated with phosphatidylinositide-3-kinase (PI3K) and mitogen-activated protein kinase (MAPK) signaling pathways under ROS activation [34,35]. In the current study, we demonstrated that KDELC2 overexpression could activate mitochondrial ROS, and it played an important role in glioblastoma neovascularization.

An inflammasome is an intracellular multi-protein complex representing the inflammatory immune response status [36]. Pattern recognition receptors (PRRs) are the basic cellular structures of the human immune system and are detected by pathogens [37]. The NOD-like receptor (NLR) proteins are intracellular components of PRRs and are divided into NLRP, NLRA, NLRB, NLRC, and NLRX [38]. Among the NLRP proteins, NLRP3 is the most well-known and is involved in the inflammatory process [38]. Persistently active NLRP3 inflammasomes are associated with some human cancers and chemoradioresistance [39]. We previously demonstrated that KDELC2 increases NF-κB expression [15]. Therefore, we first showed that KDELC2 overexpression activates the NLRP3 inflammasome via NF-κB induction and the activation of pro-ILβ and pro-caspase-1 after ROS stimulation. The detailed mechanism of how ROS induces the NLRP3 inflammasome is still unclear [40]. As applying an NLRP3 inhibitor suppressed glioblastoma angiogenesis and overexpressed KDELC2 could reverse the inhibition of tumor vascularity, we demonstrated that KDELC2 played a critical role in enhancing or regulating glioblastoma angiogenesis via ROS-induced NLRP3 activation.

Autophagy is a protein degradation process by the ubiquitin–proteasome system enabling cell survival under stress stimuli [41]. Li et al. [42] showed that autophagic inducers exhibited an anti-angiogenic effect via Beclin1, and Peg3 enhancement modulated the Wnt/β-catenin pathway and gastrin-releasing peptide and VEGFR2 expression. In contrast, starvation-induced autophagy upregulates VEGF and LC3 expression to promote endothelial cell proliferation [43]. As KDELC2 induces autophagy via upregulating LC3B, p62, and Atg4B expression and 3-MA administration suppresses HUVEC proliferation, we demonstrated that KDELC2 promotes tumor angiogenesis via increasing glioblastoma cellular autophagy. The dual autophagy roles were detected in cancer development after ROS stimulation [44]. In early cancer initiation, the formation of autophagolysosomes and the nuclear translocation of NF-E2 p45-related factor 2 effectively downregulate ROS production and prevent tumor development [45]. However, in the later stages of cancer progression and metastasis, cellular autophagy protects cancer cells from chemotherapeutically induced ROS injury and influences the TME to supply nutrients and promote aggressive behavior [46]. Kim et al. [47] showed that an increased ROS effectively induces high-grade glioma cell survival and facilitates metastasis via enhancing glycolysis and activating HIF-1α. Similarly, our results demonstrated that KDELC2-mutated glioblastoma cells could secrete angiogenic factors to stimulate endothelial cell hyperplasia via enhanced ROS-mediated HIF-1α expression and cellular autophagy.

The functions of TAM polarization are extremely diverse in cancer development. M1-macrophages play a pro-inflammatory role in abnormal phagocytic cells to inhibit tumor progression, but M2-macrophages have an anti-inflammatory role in cancer angiogenesis [48,49]. In response to cellular stress conditions, M1-macrophages produce nitric oxide synthase (NOS) and ROS and secrete IL-12 [50]. In addition, M2 macrophages secrete IL-1β, IL-10, and VEGF to induce vascular proliferation and injury repair [51]. Wang et al. [52] showed that miR-148a-3p can mediate Notch intracellular domains in TAM differentiation into M1-macrophages, which have anti-tumor properties. A hypoxic TME releases pro-angiogenic factors, including VEGF, FGF, and PDGF, resulting in tumor vascularization via the activation of IL-6 and the inhibition of IL-12 [51]. Although previous results revealed that KDELC2 increases Notch receptor expression and promotes glioblastoma progression, the detailed mechanism of KDELC2-induced glioblastoma angiogenesis is still undetermined [15]. Since tumor hypoxia plays a critical role in deciding the phenotype of TAMs and further assisting neoangiogenesis [53,54], our results implied that KDELC2 might induce tumor vascularization via the initial enhancement of HIF-1α expression, and hypoxic TME production induced ROS promotion, ER stress activation, and M2 TAM proliferation. However, direct evidence was still needed to confirm the above implications.

Ferroptosis is an iron-dependent non-apoptotic form of cell death that is stimulated by cysteine metabolism and lipid peroxidation [55]. In a recent study, ferroptosis has been found to play a pivotal role in the suppression of glioma proliferation, especially facing apoptotic resistance [56]. The elevation of intracellular ROS comprises superoxide, hydroxyl radical, hydrogen peroxide, and lipid peroxide that further induce phospholipid peroxidation and inhibit glutathione peroxidase 4 (GPX4) via serial electron transduction [57]. In addition, the ferroptosis agent promotes the p53 upregulated modulator of apoptosis (PUMA) expression that is an important mediator for the ER stress PERK–eIF2a–ATF4–CHOP pathway [58]. Furthermore, autophagy inhibits ferroptosis by resulting in ferritin degradation, but it promotes lipid peroxidation via regulated glutathione (GSH) and GPX4 [59]. Otherwise, the relationship between ferroptosis and TME had been proven to be associated with cancer progression [60]. The suppression of ferroptosis, high expression of ferroportin, and low expression of ferritin are related to the M2 polarization of TAM [61]. Therefore, since KDELC2 promotes glioblastoma ROS, ER stress, autophagy, and M2 TAM, the crosstalk between KDELC2 and ferroptosis seems relatively likely, but it needs more evidence to prove it.

## 5. Conclusions

**Figure 6:**
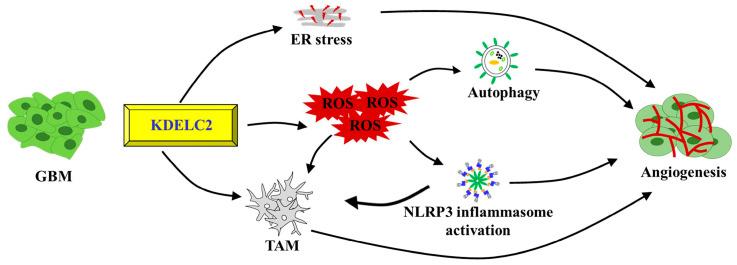
The illustration shows the mechanism of KDELC2-induced glioblastoma angiogenesis.

This study elucidated the role of KDELC2 in promoting glioblastoma angiogenesis by activating ROS, stimulating ER stress, and influencing TME (Figure 6). ROS induction resulted in NLRP3 inflammasome activation and cellular autophagy, upregulating tumor vascularization. In the future, KDELC2 might be a valuable pharmacologic target, inhibiting glioblastoma by downregulating neovascularization.

## Data Availability

Data are contained within the article.

## Supplementary Materials

The following supporting information can be downloaded at https://www.mdpi.com/article/10.3390/antiox12040923/s1, Table S1. The application of primers in qRT-PCR; Table S2. Characters of antibodies used in western blotting and IHC.
