# Supplementary material for: KDELC2 Upregulates Glioblastoma Angiogenesis via Reactive Oxygen Species Activation and Tumor-Associated Macrophage Proliferation
Source: Antioxidants (Basel). 2023 Apr 13;12(4):923. doi: 10.3390/antiox12040923 (PMC10136350; doi:10.3390/antiox12040923)
Supplement: Supplementary file 1 [file antioxidants-12-00923-s001.zip › antioxidants-2304474-supplementary.pdf]

Table. S1. The application of primers in qRT-PCR

Gene	Gene ID	Accession	Sequence	(5'->3')
KDELC2	143888	NM_001363503.2	F	TGGAGCATCGAAAAGTCAATGG
			R	CATACGTTGGAAGGACAACATCT
ZEB-2	9839	NM_014795.4	F	AAGCCAGGGACAGATCAGC
			R	GCCACACTCTGTGCATTTG
CHOP	1649	NM_001413642.1	F	CTCTGACTGGAATCTGGAGAGTG
			R	CTGAGTCATTGCCTTTCTCCTTCG
PERK	9451	NM_001313915.2	F	ACGATGAGACAGAGTTGCGAC
			R	ATCCAAGGCAGCAATTCTCCC
POFUT1	23509	NM_015352.2	F	CTGATGACCCGATGGTAAGC
			R	AAGCCTCCTTTCACCAACCT
sXBP1	22433	NM_005080.4	F	CTGAGTCCGAATCAGGTGCAG
			R	ATCCATGGGGAGATGTTCTGG
ATF4	468	NM_182810.3	F	GTTCTCCAGCGACAAGGCTA
			R	ATCCTGCTTGCTGTTGTTGG
Grp78/BiP	3309	NM_005347.5	F	TGTTCAACCAATTATCAGCAAACTC
			R	TTCTGCTGTATCCTCTTCACCAGT
EDEM1	9695	NM_014674.3	F	CAAGTGTGGGTACGCCACG
			R	AAAGAAGCTCTCCATCCGGTC
LC3B	81631	NM_022818.5	F	GATGTCCGACTTATTCGAGAGC
			R	TTGAGCTGTAAGCGCCTTCTA
ATG4b	23192	NM_178326.3	F	ATGGACGCAGCTACTCTGAC
			R	TTTTCTACCCAGTATCCAAACGG
SQSTM1	8878	NM_001142298.2	F	GCACCCCAATGTGATCTGC
			R	CGCTACACAAGTCGTAGTCTGG
CD11B	16409	NM_001145808.2	F	AAGGTGTCCACACTCCAGAAC
			R	GAGGAGCAGTTTGTTTCCAAG
CD206	17533	NM_002438.4	F	GGGAAAGGTTACCCTGGTGG
			R	TCAAGGAAGGGTCGGATCGT
TNFA	7124	NM_000594.4	F	GTGCTCCTCACCCACACCGTC
			R	CCCTTCTCCAGCTGGAAGAC
MCSF	1435	NM_000757.6	F	TCCAAAACACGGGGACCTATC
			R	TCCTCGAACACGACCACCT

Table. S2. Characters of antibodies used in western blotting and in IHC.

Antibody	Company	Host	Dilution
KDELC2	Thermo Fisher Scientific	Rabbit	1:500
Actin	Santacruz	Mouse	1:1000
NLRP3 (D4D8T)	Cell signaling	Rabbit	1:500
Caspase-1 (D7F10)	Cell signaling	Rabbit	1:500
IL-1β	R&D	Mouse	1:1000
LC3B	Sigma-Aldrich	Rabbit	1:1000
P62	Santacruz	Mouse	1:1000
VEGFR1	abcam	Rabbit	1:100
VEGFA	abcam	Mouse	1:200
